# Supplementary material for: Comparative neurotranscriptomics reveal widespread species differences associated with bonding
Source: BMC Genomics. 2021 May 31;22:399. doi: 10.1186/s12864-021-07720-0 (PMC8165761; doi:10.1186/s12864-021-07720-0)
Supplement: Supplementary file 10 — Additional file 10: Supplementary Fig. 1. Expression pattern of significant genes is consistent across regions. Plots show counts for samples collected at each pre-mating (0 h) and post-mating (0.5, 2, or 12 h) collection point. Each point represents one sample. Darker shades and triangles represent prairie vole samples. Lighter shades and circles represent meadow vole samples. a. Nfkbia in AMY (above) and VP/NAc (below). b. Pxn in HT (above) and VP/NAc (below). c. Per1 in HT (above) and VP/NAc (below). d. Cables1 in HT (above) and VP/NAc (below). Supplementary Fig. 2. Gene ontology analysis for amygdala gene-expression modules. Enriched GO terms in a. prairie vole green module and b. meadow vole darkorchid module. Hierarchical clustering tree shows relationship between GO categories based on shared genes. Branches with length of zero are subsets of one another. Fractions preceding GO terms indicate proportion of genes from the category that are included in the module of interest. FDR determined by 100 permutations where significance measures are randomly shuffled among genes. Bold text indicates adjusted p < 0.01, plain text indicates adjusted p < 0.05, and italicized text indicates adjusted p < 0.1 for term. n.s. indicates no significant terms in the category. Supplementary Fig. 3. Gene ontology analysis for hypothalamic gene-expression modules. Enriched GO terms in a. prairie vole tuquoise module and b. meadow vole seagreen module. Hierarchical clustering tree shows relationship between GO categories based on shared genes. Branches with length of zero are subsets of one another. Fractions preceding GO terms indicate proportion of genes from the category that are included in the module of interest. FDR determined by 100 permutations where significance measures are randomly shuffled among genes. Bold text indicates adjusted p < 0.01, plain text indicates adjusted p < 0.05, and italicized text indicates adjusted p < 0.1 for term. n.s. indicates no significant terms [file 12864_2021_7720_MOESM10_ESM.docx]

**SUPPLEMENTARY FIGURES**

**
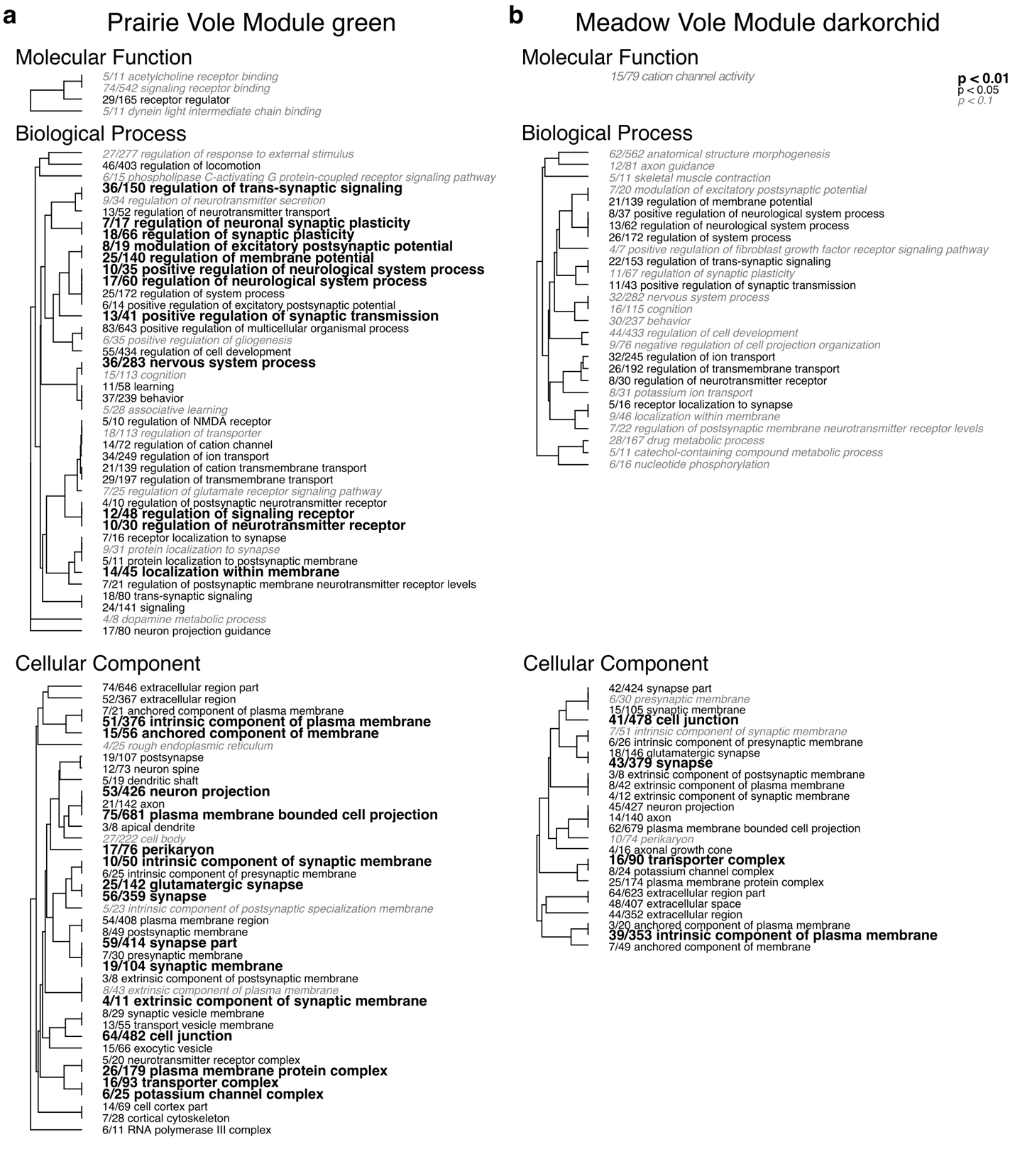
**

**Supplementary Fig. 1** Gene ontology analysis for amygdala gene-expression modules. Enriched GO terms in **a.** prairie vole green module and **b.** meadow vole darkorchid module. Hierarchical clustering tree shows relationship between GO categories based on shared genes. Branches with length of zero are subsets of one another. Fractions preceding GO terms indicate proportion of genes from the category that are included in the module of interest. FDR determined by 100 permutations where significance measures are randomly shuffled among genes. Bold text indicates adjusted p<0.01, plain text indicates adjusted p<0.05, and italicized text indicates adjusted p<0.1 for term. n.s. indicates no significant terms in the category.


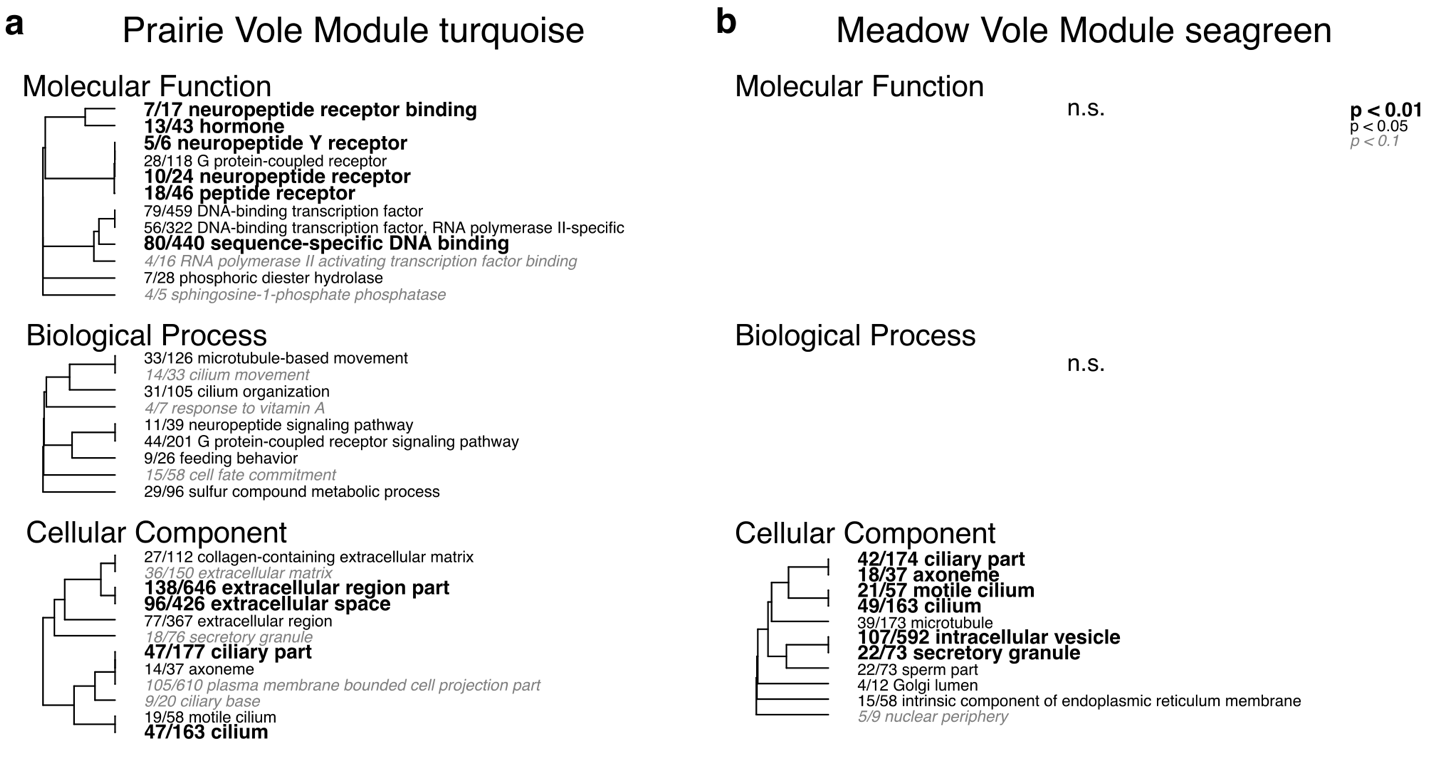


**Supplementary Fig. 2** Gene ontology analysis for hypothalamic gene-expression modules. Enriched GO terms in **a.** prairie vole tuquoise module and **b.** meadow vole seagreen module. Hierarchical clustering tree shows relationship between GO categories based on shared genes. Branches with length of zero are subsets of one another. Fractions preceding GO terms indicate proportion of genes from the category that are included in the module of interest. FDR determined by 100 permutations where significance measures are randomly shuffled among genes. Bold text indicates adjusted p<0.01, plain text indicates adjusted p<0.05, and italicized text indicates adjusted p<0.1 for term. n.s. indicates no significant terms in the category.


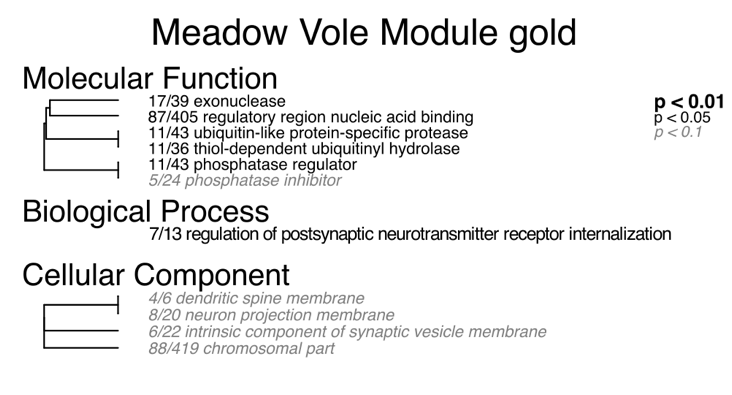


**Supplementary Fig. 3** Gene ontology analysis for ventral pallidum/nucleus accumbens gene-expression modules. Enriched GO terms in meadow vole gold module. (There were no significant enrichments for the prairie vole magenta module.) Hierarchical clustering tree shows relationship between GO categories based on shared genes. Branches with length of zero are subsets of one another. Fractions preceding GO terms indicate proportion of genes from the category that are included in the module of interest. FDR determined by 100 permutations where significance measures are randomly shuffled among genes. Bold text indicates adjusted p<0.01, plain text indicates adjusted p<0.05, and italicized text indicates adjusted p<0.1 for term. n.s. indicates no significant terms in the category.


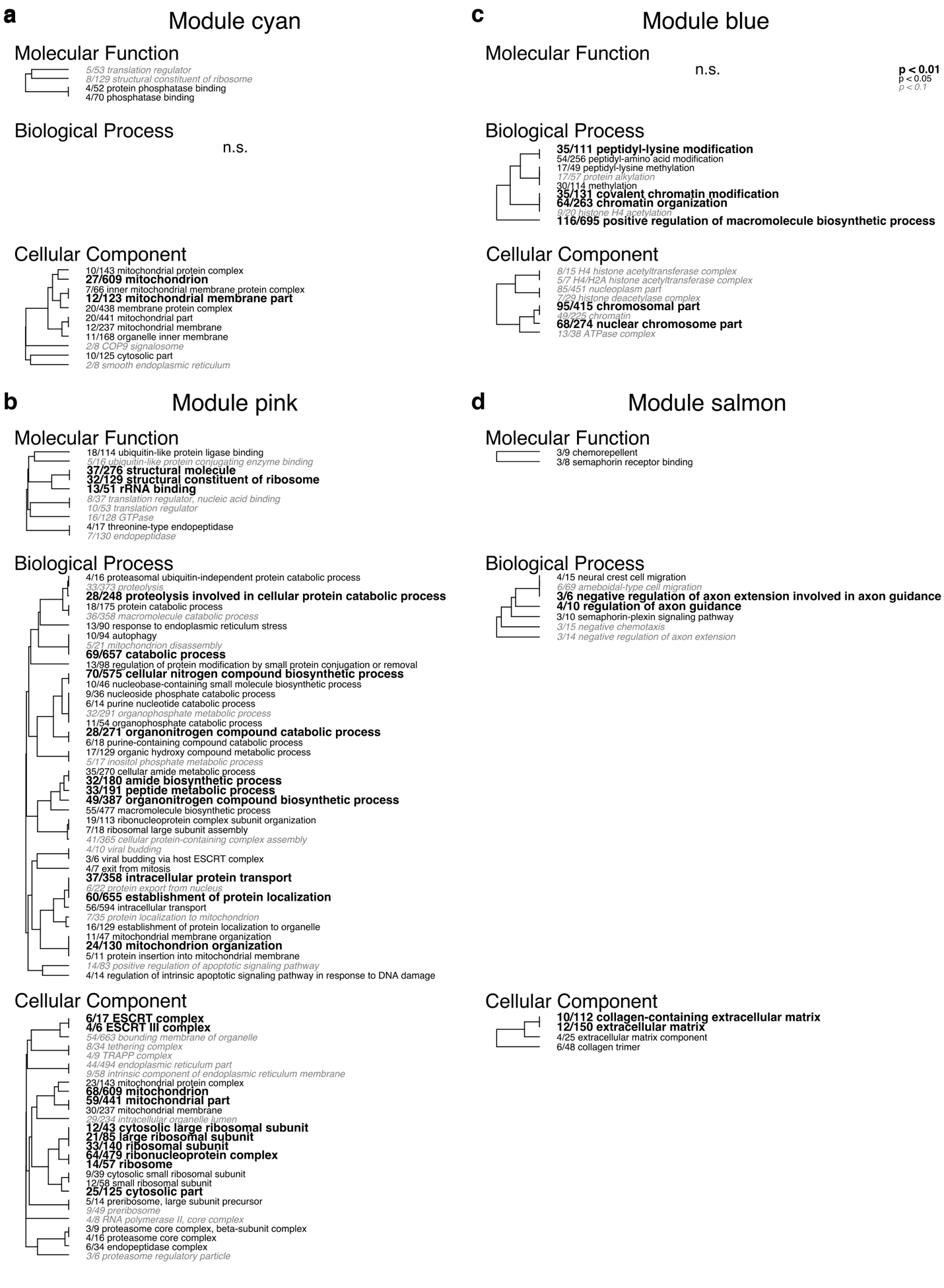


**Supplementary Fig. 4** Effects of mating on gene ontology categories. Enriched GO terms in modules most strongly positively (**a-b**) and negatively (**c-d**) correlated with mating status in prairie voles. **a.** cyan module, **b.** pink module **c.** blue module, **d.** salmon module. Hierarchical clustering tree shows relationship between GO categories based on shared genes. Branches with length of zero are subsets of one another. Fractions preceding GO terms indicate proportion of genes from the category that are included in the module of interest. FDR determined by 100 permutations where significance measures are randomly shuffled among genes. Bold text indicates adjusted p<0.01, plain text indicates adjusted p<0.05, and italicized text indicates adjusted p<0.1 for term. n.s. indicates no significant terms in the category.
